# The Mental Health Implications of Informal Care Receipt Stability

**DOI:** 10.1093/geroni/igaf122.1120

**Published:** 2025-12-31

**Authors:** Yezhen Li, Marco Angrisani, Jinkook Lee

**Affiliations:** University of Southern California, Los Angeles, California, United States; University of Southern California, Los Angeles, California, United States; University of Southern California, Los Angeles, California, United States

## Abstract

Scholars have shown a growing interest in how informal care receipt shapes older adults’ mental health outcomes. However, little is known about the longitudinal dynamics of informal care receipt - specifically, the stability of informal care - and their contributions to care recipients’ psychological well-being. Using data from the 2010-2018 waves of the Health and Retirement Study (n = 4,160 respondents; 8,332 person-year observations), this study employs mixed-effect regressions to investigate how informal care receipt stability predicts depressive symptoms among older adults with persistent care needs, and how it buffers the stress from activities of daily living (ADL) and instrumental activities of daily living (IADL) limitations. Results suggest that receiving stable informal care, including persistent care (b = -.345; 95% CI = -.481, -.209) and partial loss of informal care sources (b = -.320; 95% CI = -.515, -.125), is significantly associated with lower depressive symptoms than not receiving informal care. Stable informal care receipt also weakens the association between IADL limitations and depressive symptoms through moderation effects, suggesting a stress-buffering pattern, though it does not play a similar role against ADL limitations. In contrast, unstable informal care receipt, including total care loss and caregiver transition, is not associated with any mental health benefits. The findings imply that informal care stability may serve as a measure of care quality through a longitudinal lens, and policies encouraging caregiving commitments may be crucial for the mental health of long-term care recipients.

